# Genotypic trade-off between appetitive and aversive capacities in honeybees

**DOI:** 10.1038/s41598-019-46482-4

**Published:** 2019-07-16

**Authors:** Pierre Junca, Lionel Garnery, Jean-Christophe Sandoz

**Affiliations:** Evolution, Genomes, Behavior and Ecology, CNRS, Univ Paris-Sud, IRD (UMR 9191), 1 avenue de la Terrasse, Gif-sur-Yvette, 91198 France

**Keywords:** Haplotypes, Classical conditioning

## Abstract

Honey bees can learn both appetitive and aversive associations, using two olfactory conditioning protocols. Appetitive conditioning of the proboscis extension response (PER) involves associating an odor, the conditioned stimulus (CS) with a sucrose solution, the unconditioned stimulus (US). Conversely, aversive conditioning of the sting extension response (SER) involves associating the odor CS with an electric or thermal shock US. Here, we investigated the relationship between bees’ appetitive and aversive learning capacities at the individual level and the influence of bees’ genotype. As learning performance was shown to depend on an individuals’ sensitivity to the US, we systematically measured four different traits in each individual bee: sensitivity to sucrose, PER learning performance with a sucrose US, sensitivity to temperature, SER learning with a temperature US. First, we confirmed for both conditioning types that learning performance correlates with US responsiveness. Second, we found a trade-off between appetitive and aversive learning performances: bees that were better appetitive learners (and had a lower sucrose US threshold) learned less efficiently in the aversive conditioning (and had a higher temperature US threshold). Because the honey bee queen typically mates with 15–20 males, the workers from a honey bee hive belong to as many different patrilines, allowing for the search of the genetic determinism of cognitive abilities. Using microsatellite analysis, we show that a genetic determinism underlies the trade-off between appetitive and aversive capacities, with appetitively *vs* aversively biased patrilines. The honey bee hive thus appears as a genetically structured cognitive community.

## Introduction

Where to find food and how to avoid danger? These are two simple but critical questions animals need to answer for surviving in a wild environment. Individual experience plays a major role in solving these questions, since animals can learn to associate initially neutral environmental stimuli (odors, sounds, colors, etc.) with their upcoming consequences, both beneficial (appetitive) and noxious (aversive). Therefore, an important part of an individual’s potential fitness resides in its genetically-determined appetitive and aversive learning abilities. This is particularly true for solitary species, in which individuals must be skilled in both types of tasks since they must provide alone for all of their needs. The emergence of sociality, multiple times in the course of evolution, has fundamentally changed this rule, because in a social group different abilities may be distributed among different members, giving rise to behavioral specialization^1^. Such inter-individual differences are thought to be beneficial for a social group’s ecological success. In meerkats, for instance, particular individuals in the group are dedicated to the surveillance of the surroundings while others take care of the youth and still others forage for the group^2,3^. In noisy miners, different birds specialize in either defense against predators or in provisioning^4^. Such behavioral specialization is even more conspicuous within social insect colonies, where division of labor among non-reproductive individuals is a hallmark of social lifestyle^5–7^. At the proximal level, division of labor is commonly explained through self-organization based on individual behavioral rules that rely on inter-individual differences in responses to environmental stimuli^7,8^. The fixed-threshold model, in particular, assumes that specialization in a social group arises spontaneously from differences among individuals in their response threshold to stimuli associated with specific tasks^9–11^. Generally, individuals with the lowest threshold will engage in the corresponding task, provoking a reduction in the intensity of the task-associated stimulus. Division of labor may thus appear through simple inter-individual differences in the response threshold to different signals.

Response thresholds do not only influence the propensity of individuals to perform a specific task, but they also control associative learning performances, as shown for both aversive and appetitive modalities in the honeybee *Apis mellifera*. In the appetitive conditioning of the Proboscis Extension Response (PER)^12,13^, in which bees have to associate an odor with a sucrose reward, learning performances are strongly under the influence of individual response thresholds to sucrose^14,15^. Thus, bees that are more sensitive (i.e. show a higher responsiveness) to sucrose display higher learning performances when associating an odor with sucrose. Likewise, in the aversive conditioning of the Sting Extension Response (SER), in which bees have to associate an odor with an electric shock or heat punishment^16,17^, learning performances are directly correlated with an individual’s responsiveness to the aversive reinforcer (electric shock^18^; heat^16^). The self-organization theoretical account presented above predicts that within a social group, different individuals should display different response thresholds to appetitive and aversive stimuli, as they are related to different tasks, respectively food-associated tasks and defense-oriented tasks. Interestingly, at the population level, a trade-off has been observed between a hives’ foraging activity and its defensive ability^19^. Hives with a high foraging activity displayed low defense responses and vice versa. As this trade-off is thought to rely on a genetic background, one could expect to find a similar trade-off in individuals’ aversive and appetitive abilities. While some individuals would be biased towards appetitive abilities (and would be comparably less skilled for aversive tasks) other individuals would be biased towards aversive abilities. This attractive hypothesis has seldom been tested directly and no demonstration of its validity exists yet.

In honeybees, numerous studies have led to the idea that bees’ sensitivity to sucrose was the main determinant of task allocation^14,20–22^. Evidence showing that sucrose responsiveness correlates with responsiveness to a number of other sensory stimuli initially supported this idea (e.g. tactile^23^; light^24^). However, the stimuli tested in these studies were mostly connected to foraging-related tasks. More recently, Roussel *et al*.^18^ compared bees’ responsiveness to sucrose with responsiveness to a stimulus unrelated to foraging, but rather belonging to the aversive hedonic modality: an electric shock. This study reported that sucrose responsiveness and electric shock responsiveness are not correlated, suggesting the existence of other determinants to bees’ behavior^18^. This study concluded that appetitive and aversive sensitivities belong to two independent behavioral modules, associated respectively to foraging-related and defense-related tasks. The lack of correlation observed by Roussel *et al*. could be taken for an invalidation of the hypothesis of a trade-off between appetitive and aversive abilities proposed above. However, these experiments were carried out on individuals of unknown age, which may have added a confounding variable in the analysis. Indeed, the sucrose response threshold varies with the bees’ age^21,25,26^ as does their sensitivity to aversive stimuli (electric shock^27,28^). Therefore, controlling the bees’ age may be critical for unraveling potential appetitive vs aversive trade-offs among individuals.

A major question that arises from threshold models of self-organization and the data presented above concerns the genetic substrate underlying such differences in sensory thresholds among individuals. The monogynous and polyandrous reproductive system of honeybees provides a good opportunity for studying this question. In a honeybee colony, the diploid queen mates on average with 15–20 haploid males^29^. Therefore, the workers, her daughters, belong to as many different patrilines with different genetic backgrounds within the hive. Workers’ patriline origin has an impact on task allocation as observed on brood care, foraging and defensive behavior^30^. In addition, it is known to have an impact on sensory responsiveness and learning performances. In the aversive modality, we showed previously that bees from different patrilines have different thermal response thresholds and show accordingly different aversive learning performances with this reinforcement^16^. In the appetitive modality, differences in learning performances among patrilines are suspected^31^, especially because sucrose response thresholds vary among them^32^. So far, the study of genotypic determinism on responsiveness and learning has been studied independently within the appetitive or within the aversive modality. Therefore, a possible trade-off in aversive vs appetitive learning abilities among different patrilines is utterly unknown.

In the present study, we asked how sensitivity and learning capacity in appetitive and aversive modalities are distributed among individuals composing a honeybee colony, in particular with regards to their patriline of origin. Performing the experiments on age-controlled individuals, we found a clear trade-off between aversive and appetitive abilities at the individual level. This aversive *vs* appetitive trade-off appeared also when taking into account the bees’ patrilines. These results suggest that within a eusocial insect colony workers compose an equilibrium of cognitively-specialized individuals, giving rise to a complex but highly-adaptable cognitive community.

## Results

To assess how appetitive and aversive sensitivities and learning performances are related, series of four experiments were carried out on age-controlled (two weeks old) honey bee workers. Half of the bees went through an appetitive evaluation day followed by an aversive one, and the other half underwent the reversed schedule. The appetitive evaluation day comprised a *sucrose responsiveness* procedure followed by a *PER conditioning* procedure. Analogously, the aversive evaluation day comprised a *heat responsiveness* procedure followed by a thermal *SER conditioning* procedure. In the *responsiveness* procedures, bees received appetitive (sucrose) or aversive (temperature) stimuli of increasing intensity alternated with control stimulations (water and tactile respectively). In the *conditioning* procedures, bees were subjected to a differential conditioning protocol in which they had to differentiate between a reinforced odor (CS+) and a non-reinforced odor (CS−). For appetitive learning, the CS+ was associated with a sucrose reward and for aversive learning, the CS+ was associated with a temperature punishment. Bees received 8 CS +and 8 CS− trials in a pseudorandomized order with 10 min inter-trial intervals. For appetitive procedures, the bees’ PER were measured, while for aversive procedures, the SER were measured.

### Responsiveness to appetitive and aversive stimulations

**Figure 1:**
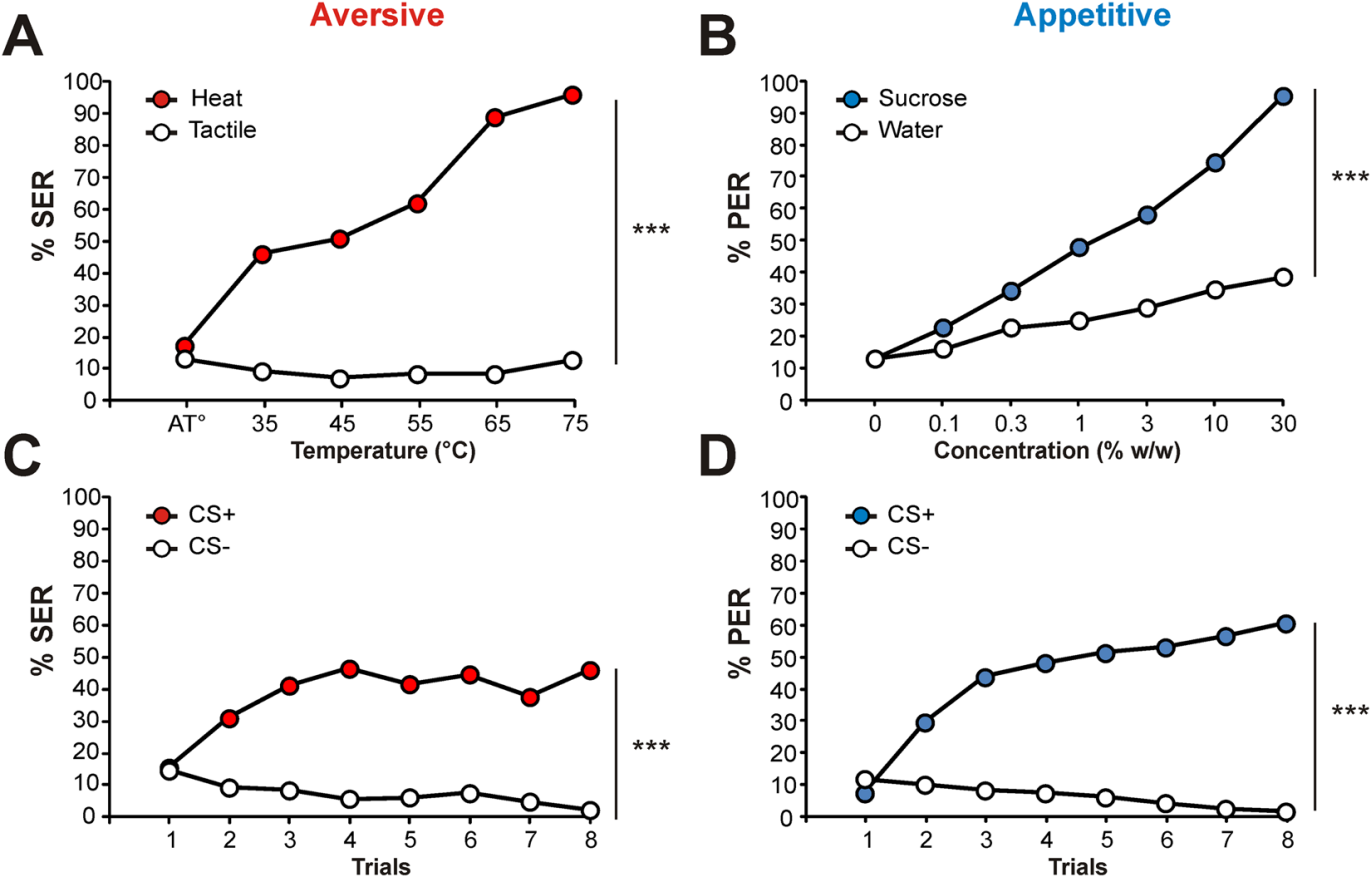
Responsiveness and learning protocols for appetitive and aversive hedonic modalities performed on the same individuals (n = 226). (**A**) Heat responsiveness. Red circles, %SER to a series of increasing temperatures; white circles, %SER of the same bees to the presentation of a tactile (unheated) stimulus (control). (**B**) Sucrose responsiveness. Blue circles, %PER to a series of sucrose solutions of increasing concentration; white circles, %PER of the same bees to the presentation of water (control). (**C**) Differential aversive conditioning of the SER. Red circles, %SER to the CS+ (reinforced odor) along the 8 trials; white circles, %SER to the CS− (non-reinforced odor). (**D**) Differential appetitive conditioning of the PER. Blue circles, %PER to the CS+ along the 8 trials; white circles, %PER to the CS−. (ANOVA for repeated measurements, AB; stimulus x concentration or CD: stimulus x trial interaction, ***p < 0.001).

In the *heat responsiveness* experiment (Fig. 1A), bees’ SER increased significantly with increasing temperature (from 17% to 96%, ANOVA for repeated measurements: F_5,1125_ = 148.7, p < 0.001). In contrast, responses to alternated tactile stimulus applications remained stable throughout the experiment (between 12% and 13%, F_5,1125_ = 2.07, NS). Accordingly, responses to heat stimuli increased more quickly than control stimulations throughout the procedure (*stimulus* x *trial* interaction, F_5,1125_ = 106.5, p < 0.001). In the *sucrose responsiveness* experiment (Fig. 1B), bees’ PER increased significantly with increasing sucrose concentration (from 13% to 95%, F_6,1350_ = 180.1, p < 0.001. A response increase was also noticed in the control water stimulations (from 13% to 38%, F_6,1350_ = 24.7, p < 0.001) but on a smaller scale. This increase in water responses can be attributed to a non-associative sensitization effect which probably built up in the course of the procedure^33^. In any case, sucrose responses increased more quickly that control stimulations throughout the experiment (*stimulus* x *trial* interaction, F_6,1350_ = 59.5, p < 0.001).

### Appetitive and aversive conditioning performances

Bees learned both appetitive and aversive tasks effectively. In the aversive learning protocol (Fig. 1C), bees’ SER to the reinforced (CS+) odorant increased significantly (from 15% to 46%, F_7,1575_ = 20.8, p < 0.001), while their responses to the non-reinforced odorant (CS−) decreased (F_7, 1575_ = 5.87, p < 0.001). Consequently, bees’ responses to the CS+and CS− developed differently (*stimulus* x *trial* interaction: F_7,1575_ = 25.8, p < 0.001). In the appetitive learning protocol (Fig. 1D), bees’ PER to the CS+ increased along trials (from 8% to 61%, F_7,1575_ = 98.7, p < 0.001) while responses to the CS− decreased (F_7,1575_ = 7.80, p < 0.001). Overall, bees managed to differentiate between the two conditioned stimuli (*stimulus* x *trial* interaction: F_7,1575_ = 91.1, p < 0.001). Data obtained in responsiveness and learning experiments for aversive and appetitive modalities were consistent with previous studies performed separately on these two modalities^16,34^.

### Appetitive and aversive relationships at the individual level

**Figure 2:**
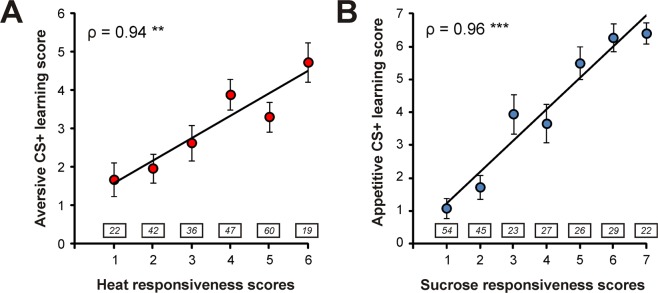
Relationship between responsiveness and learning performances within appetitive and aversive modalities. (**A**) Correlation between heat responsiveness scores and aversive learning scores in the same bees. Individuals were grouped according to their heat responsiveness scores. (**B**) Correlation between sucrose responsiveness scores and appetitive learning performance scores in the same bees. Individuals were grouped according to their sucrose responsiveness score. Numbers of individuals are indicated in boxes above each category (Spearman correlations, **p < 0.01; ***p < 0.001, n_tot_ = 226, mean ± SEM).

To study the relationships between responsiveness and learning performances within each hedonic modality or between the two modalities, we calculated individual scores^16,18^. *Responsiveness* scores consisted in the sum of responses to sucrose stimuli or to heat stimuli in each procedure. Similarly, learning scores were calculated as the sum of PER or SER responses to the CS+ along trials for appetitive and aversive learning protocols respectively. Previous work showed clearly that individual learning performance and responsiveness to the reinforcing stimulus are strongly correlated both in the aversive modality (electric shock^18^; heat^16^) and in the appetitive modality (sucrose^14,34,35^). But are these relationships noticeable when experiments are performed on the same individuals? In full agreement with previous work, we found strong and significant correlations between heat responsiveness and aversive learning performance (Fig. 2A; Spearman correlation, ρ = 0.94, p < 0.01) and between sucrose responsiveness and appetitive learning performance (Fig. 2B; ρ = 0.96, p < 0.001).

**Figure 3:**
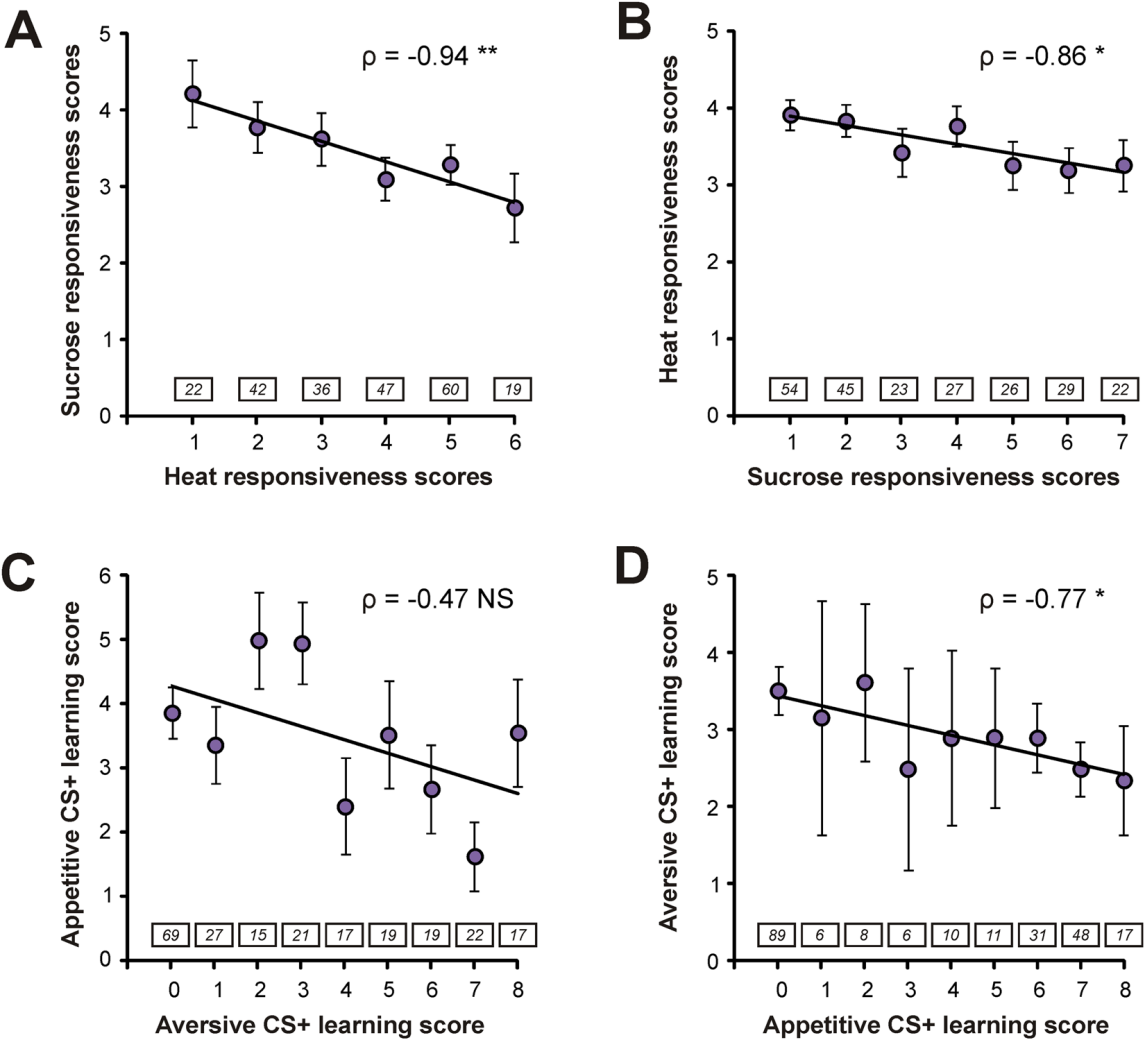
Relationship between appetitive and aversive performances at the individual level. (**A**,**B**) Relationship between heat responsiveness and sucrose responsiveness scores. Bees were grouped according to either heat responsiveness scores (**A**) or sucrose responsiveness scores (**B**). (**C**,**D**) Relationship between appetitive and aversive learning scores. Bees were grouped according to either aversive learning scores (**C**) or appetitive learning score (**D**). Numbers of individuals are indicated in boxes above each category (Spearman correlations, *p < 0.05; **p < 0.01, NS: not significant; n_tot_ = 226, mean ± SEM).

Measuring appetitive and aversive scores in the same individuals provided us with the opportunity to compare responsiveness and learning performances between hedonic modalities. To do that, individual bees can be grouped either according to aversive scores or to appetitive scores. Figure 3 presents both possibilities (aversive grouping: Fig. 3A–C; appetitive grouping: Fig. 3B–D). We found a clear negative correlation between appetitive and aversive *responsiveness* scores, which was present both when grouping individuals according to heat responsiveness scores (Fig. 3A: ρ = −0.94; p < 0.01) or to sucrose responsiveness scores (Fig. 3B: ρ = −0.86; p < 0.05). When comparing appetitive and aversive *learning* scores, we observed a significant negative correlation when grouping individuals according to appetitive learning scores (Fig. 3D: ρ = −0.77; p < 0.05) but the relation was not significant when grouping bees according to aversive learning scores (Fig. 3C: ρ = −0.47; p = 0.21).

**Figure 4:**
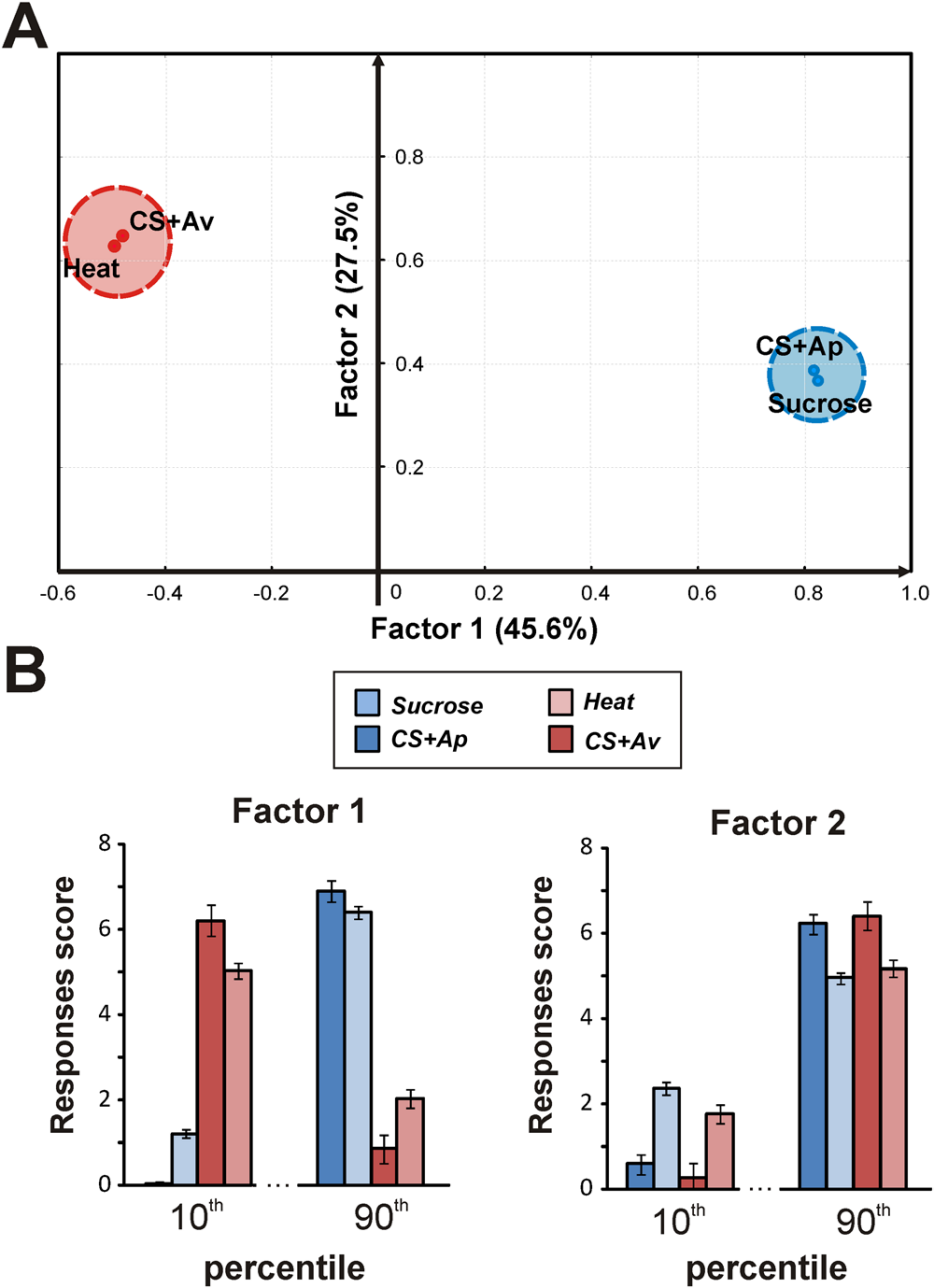
Factor analysis on appetitive and aversive performances. (**A**) factor analysis on the 4 response scores (*Sucrose*: sucrose responsiveness; *CS*+*Ap*: appetitive learning score; *Heat*: heat responsiveness; *CS*+*Av*: aversive learning score) measured in 226 individuals. Two main factors with eigenvalues higher than 1 are extracted. Factor 1 (45.6% variance) shows a clear opposite relationship between appetitive and aversive variables. Factor 2 (27.5% variance) is related to differences in average response magnitude among individuals. (**B**) Response scores (mean ± SEM) of the first and last 10% of the distribution of individuals on Factor 1 or Factor 2.

The grouping of individual responses according to the each type of score, as performed above, produced in some cases uneven data distributions (from 6 to 89 bees per group) which could make correlation analyses tricky. To avoid this problem, a Factor Analysis (FA), which does not require grouping data according to one or the other modality, was performed (Fig. 4A). The four variables (heat responsiveness, sucrose responsiveness, appetitive learning and aversive learning scores) were best explained by 2 main factors (73.1% of overall variance). Factor 1 (45.6% overall variance) clearly segregated the hedonic modalities, with appetitive responsiveness and learning scores corresponding to positive values on Factor 1 and aversive scores corresponding to negative values. The coordinates of individual bees on this axis correlated positively with appetitive variables (responsiveness: ρ = 0.63; p < 0.001; learning: ρ = 0.60; p < 0.001) and negatively with aversive variables (responsiveness: ρ = −0.37; p < 0.001; learning: ρ = −0.30; p < 0.001). Accordingly, the bees that had the lowest loading on Factor 1 (<10^th^ percentile) showed high aversive scores and weak appetitive scores (Fig. 4B). Conversely, bees that had the highest loading on Factor 1 (>90^th^ percentile) showed high appetitive scores and weak aversive scores.

Factor 2 (27.5% variance) was positively correlated with both aversive and appetitive modalities (aversive responsiveness: ρ = 0.49; p < 0.001; aversive learning: ρ = 0.55; p < 0.001; appetitive responsiveness: ρ = 0.24; p < 0.001; appetitive learning: ρ = 0.27; p < 0.001), and represented the general response magnitude of bees over all scores. Thus, bees below the 10^th^ percentile on Factor 2 showed generally low scores while bees above the 90^th^ percentile displayed high scores. This analysis shows that bees’ behavior could be defined primarily as a hedonic bias (Factor 1) and secondarily as a general response magnitude (Factor 2). These data thus demonstrate the opposite relationship existing at the individual level between appetitive and aversive performances. We next evaluated whether these relationships rely on a genotypic determinism.

### Appetitive and aversive learning at the patriline level

**Figure 5:**
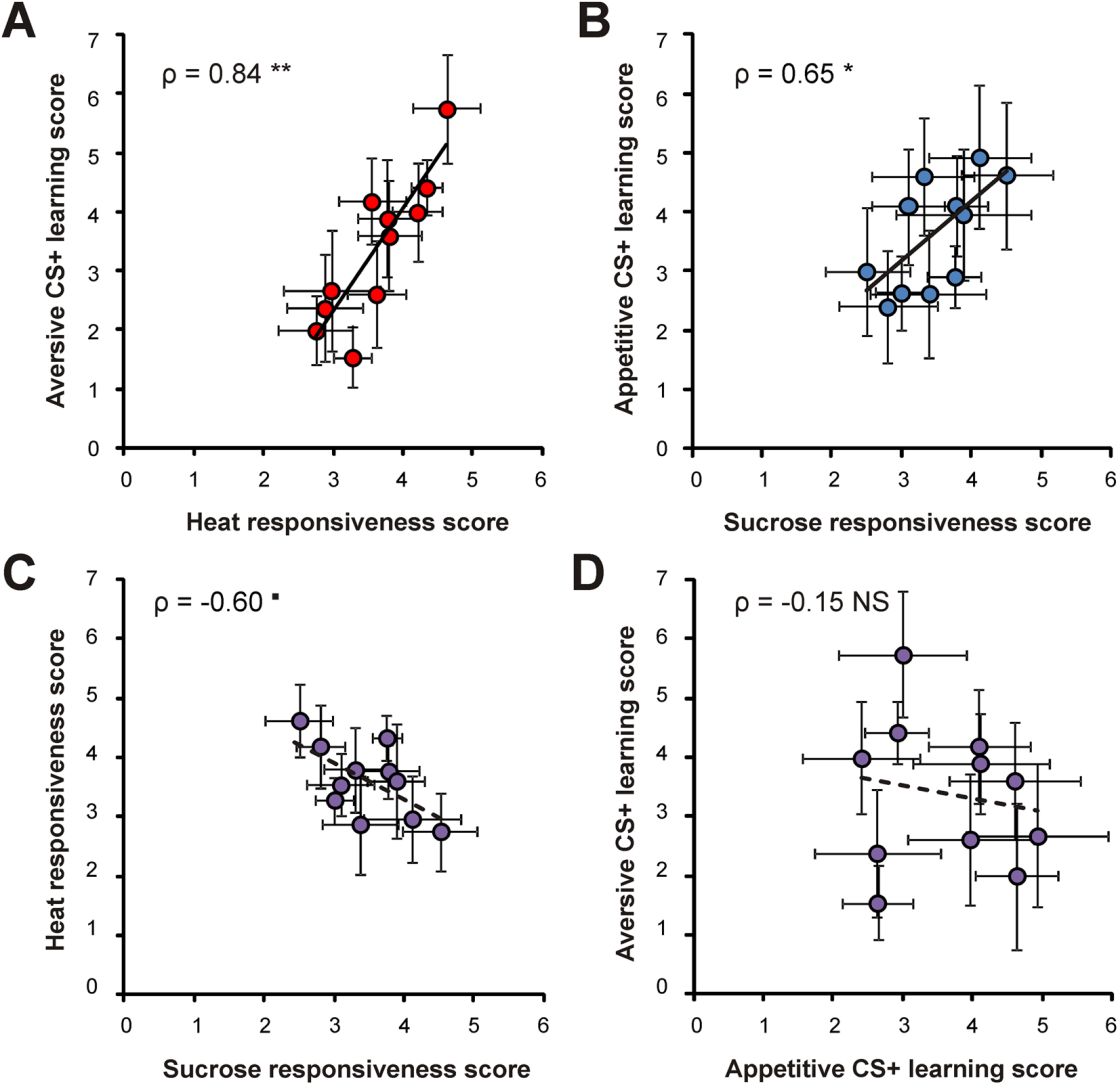
Patriline influence on the relationship between aversive and appetitive performances. Individual scores are grouped according to each worker’s patriline. (**A**) Correlation between heat responsiveness and aversive learning scores among patrilines. (**B**) Correlation between sucrose responsiveness and appetitive learning performance scores among patrilines. (**C**) Relationship between heat responsiveness and sucrose responsiveness scores at the patriline level. (**D**) Relationship between appetitive and aversive learning performance scores at the patriline level. (Spearman correlations, ^▪^p = 0.067, *p < 0.05, ***p < 0.01, NS: not significant; n = 11 patrilines, 9 df, mean ± SEM).

To evaluate whether responsiveness and learning performance relationships are influenced by the bees’ genotype, we used a microsatellite analysis and determined each worker’s patriline. From the initial 226 individuals from 2 colonies, we obtained 25 patrilines containing between 3 and 28 individuals. For assessing patriline performance scores accurately, we only used data from the 11 patrilines which contained more than 8 individual bees. The bees’ responsiveness and learning scores in both modalities were pooled according to each worker’s patriline (Fig. 5). Within each modality, we found that patrilines that were highly responsive to thermal stimuli also presented high aversive learning performances and *vice versa* (Fig. 5A; ρ = 0.84; p < 0.01). Similarly, patrilines with a high sucrose responsiveness score presented a high appetitive learning score and *vice versa* (Fig. 5B; ρ = 0.65; p < 0.05). This confirms at the genotype level, the relationships observed above between responsiveness and learning within each modality.

When correlations were performed across aversive and appetitive modalities, we noticed a difference with observations at the individual level (Fig. 3C,D). Thus, the negative relationship between heat responsiveness and sucrose responsiveness scores was only near significant (Fig. 5C; ρ = −0.60, p = 0.067). Moreover, aversive and appetitive learning showed a rather scattered relationship and the correlation coefficient was not significant (Fig. 5D; ρ = −0.15; NS).

**Figure 6:**
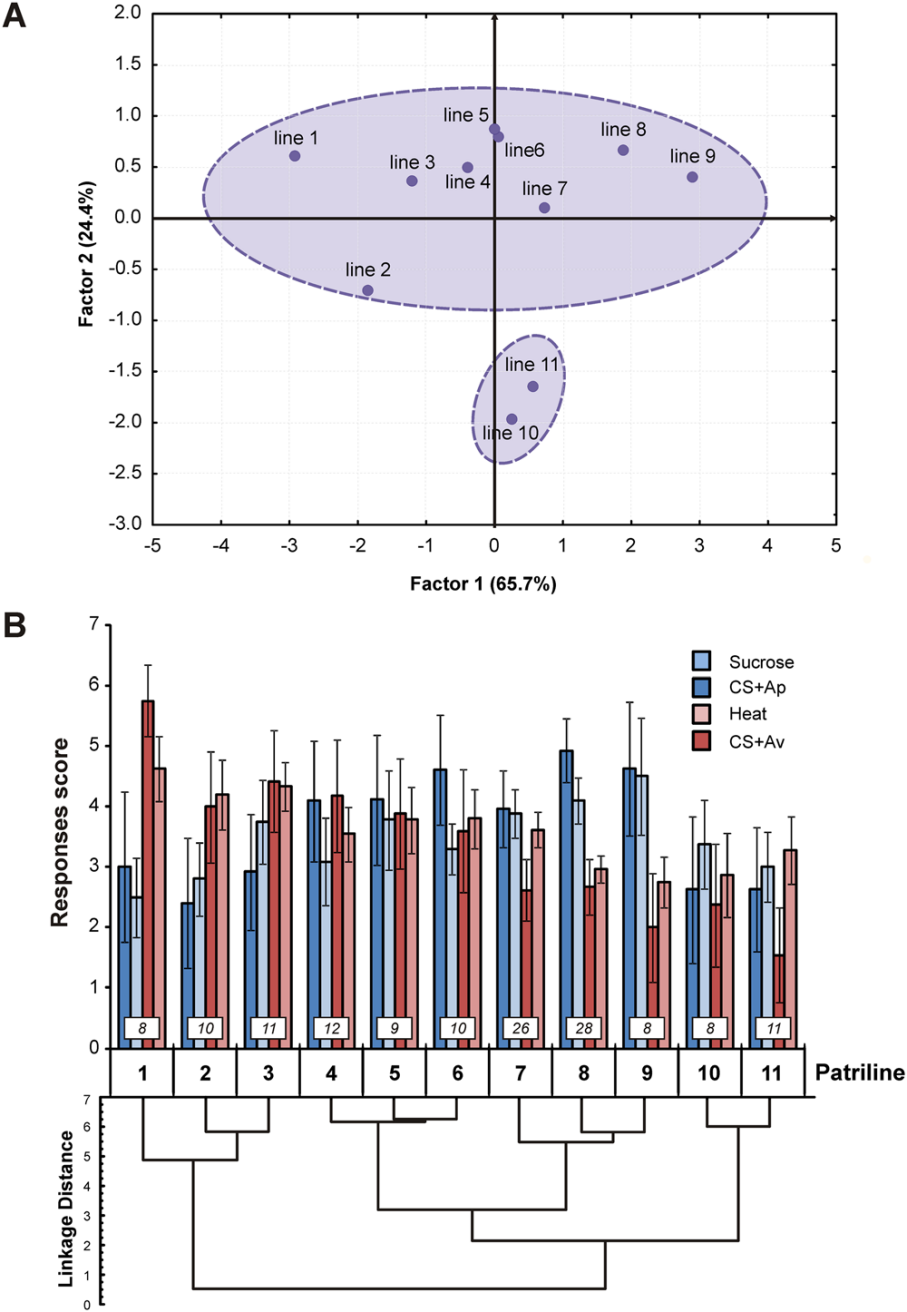
Multivariate analyses of appetitive and aversive performances at the patriline level. (**A**) Factor analysis presenting each patriline according to 2 main factors. Factor 1 is a hedonic bias factor, equivalent to Factor 1 in Fig. 4A. Patrilines on the left show high response scores in aversive procedures, while patrilines on the right display stronger appetitive performances. Two patrilines contribute significantly to Factor 2 and exhibit weak scores in both appetitive and aversive procedures. (**B**) Hierarchical clustering dendrogram (Ward’s method) showing for each patriline its average performance score (mean ± SEM): sucrose responsiveness (light blue), appetitive learning (CS+ Ap, dark blue), heat responsiveness (light red), aversive learning (CS+ Av, dark red). Numbers of individuals are indicated in boxes above each patriline.

We reasoned that such apparent lack of consistency between data at the individual and at the patriline level (Figs 3C,D vs 5C,D) may be explained by some patrilines behaving very differently from the rest. To understand this phenomenon, we subjected the patriline data to a factor analysis (FA) and to a cluster analysis (Fig. 6). These analyses confirmed our hypothesis and indicated the existence of two subgroups. First, the factor analysis extracted two main factors (Fig. 6A, 90.1% of overall variance), which were the same factors that appeared at the individual level (Fig. 4A). Factor 1 (65.7% variance) represented the hedonic bias, patrilines exhibiting high performances in aversive procedures and weak performances in appetitive procedures being located on the left of this axis and *vice versa* for patrilines located on the right (compare with Fig. 6B). As above, Factor 2 (24.4% variance) represented general response magnitude.

**Figure 7:**
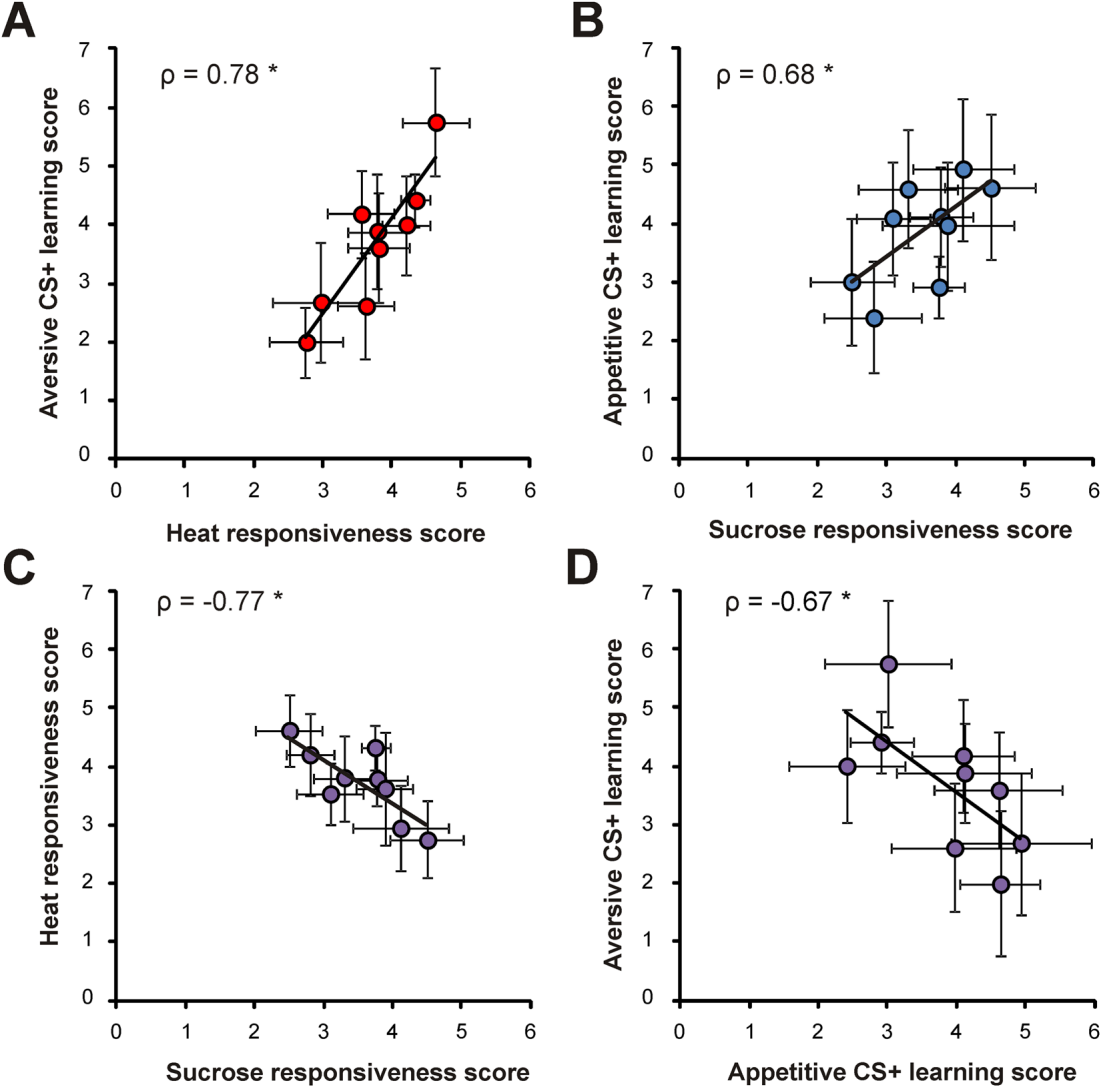
Patriline influence on the relationship between aversive and appetitive performances, without low-score patrilines. Individual scores are grouped according to each worker’s patriline. (**A**) Correlation between heat responsiveness and aversive learning performance scores among patrilines. (**B**) Correlation between sucrose responsiveness and appetitive learning performance scores among patrilines. (**C**) Correlation between heat responsiveness and sucrose responsiveness scores. (**D**) Correlation between appetitive and aversive learning performance scores. (Spearman correlations, *p < 0.05; n = 9 patrilines, 7 df, mean ± SEM).

Two patrilines with generally low scores (lines 10 and 11) contributed 66.7% to this Factor, and were segregated from the other patrilines (lines 1 to 9). These two patrilines were also clearly segregated by the cluster analysis (Fig. 6B). As they did not follow the general response pattern, we further evaluated the relationship between aversive and appetitive scores without the contribution of patrilines 10 and 11. This data selection did not modify the positive relationships between responsiveness and learning (Fig. 7A,B; aversive: ρ = 0.78; p < 0.05; appetitive: ρ = 0.68; p < 0.05). However, it allowed demonstrating at the patriline level the negative correlation existing between appetitive and aversive modalities. Thus, heat responsiveness was negatively correlated to sucrose responsiveness (Fig. 7C; ρ = −0.77; p < 0.05) and aversive learning performance was negatively correlated to that in appetitive learning (Fig. 7D; ρ = −0.67; p < 0.05). These negative correlations between hedonic modalities support the idea of some genetic specialization of patrilines in either appetitive or aversive abilities.

## Discussion

In this study, we assessed responsiveness and learning performance in both appetitive and aversive hedonic modalities on the same, age-controlled, individuals. At the individual level, we confirmed within each modality that responsiveness to a given reinforcement (sucrose or heat) determines learning performance with this reinforcement (PER conditioning or SER conditioning). Moreover, we found a trade-off between appetitive and aversive modalities, so that performances within one modality were negatively correlated with those in the other. Using microsatellite analysis, we confirmed both within-modality and between-modality relationships on a patriline level, thus demonstrating a genetic influence on the appetitive/aversive trade-off. Our data also show that a low proportion of the patrilines displays generally low scores in both hedonic modalities and do not follow the general trade-off.

### A hedonic trade-off within the hive

Our results, both at the individual and at the patriline level demonstrate the existence of a trade-off between bees’ appetitive and aversive abilities. This result suggests that the honeybee colony is a cognitive community composed of specialized individuals displaying an appetitive or an aversive bias. The idea of possible interactions between appetitive and aversive skills in worker bees has been suggested early on because opposite tasks like foraging and colony defense are both undertaken by older bees^36^. Thus, according to the response threshold model, there should be differences among older bees in response thresholds to stimuli associated with each type of task. Our results obtained on 2 weeks old individuals, an age at which foragers may already engage in foraging or guarding^37,38^, provide explicit support for this idea. One needs however to remain cautious when predicting how this trade-off will affect a real hive. First, we took a snapshot at 2 weeks of age and do not know how well this trade-off is maintained throughout the bees’ lifetime. In a real colony, workers of all ages interact, which may makes the situation difficult to predict. Second, how this trade-off may translate into actual task allocation within the hive is not trivial. Previous studies already demonstrated that nectar foragers and guards differ in their responsiveness to both sucrose^39^ and electric shock (^18^, equivalent to our heat stimulus). One could thus expect our bees with different hedonic biases to engage in different tasks, for instance foraging and guarding. However, direct predictions are difficult because task allocation is under the control of many environmental variables, including colony size, time of year, climatic conditions and food availability^5^. In addition, the observed relationship between sensory responsiveness and performed task does not appear to be as simple as stated by response threshold models. For instance, contrary to the prediction of these models, nectar foragers were found to be less sensitive to sucrose than guards^39^, while guards are less sensitive to the electric shock than foragers^18^. Indeed, nectar foragers and guards are particularly selective with regards to the stimulus intensities to which they should respond in nature, instead of being more sensitive. Such high selectivity may be adaptive for honeybees, when taking costs and benefits for the colony into account: nectar foragers would optimize this ratio by compensating the high flying costs by gathering only nectar at the highest sugar concentrations, while guards would optimize this ratio by triggering costly defensive responses only to the strongest aggressions^18,22^. Given this apparent inconsistency between the predictions of threshold based models and task allocation in honeybees, it will be especially important to relate in future work the hedonic bias we have shown here with the actual propensity of workers to engage in foraging or guarding tasks.

### Inter-individual differences in appetitive and aversive sensitivities

Our data show genetically-determined inter-individual differences in the bees’ sensitivity to sucrose and thermal stimuli, which translate into differences in learning performances in both modalities. These discrepancies in sensory sensitivity between individuals may be based on neuroanatomical and/or neurophysiological differences and could involve multiple levels of the respective sensory pathways, from receptors at the periphery until neural circuits in the brain. Inter-individual differences in sucrose sensitivity, for instance, could happen because of different numbers and/or affinities of sucrose (AmGr1) receptors within gustatory neurons; different numbers of gustatory neurons present on the bees’ antennae; different numbers of synaptic contacts of gustatory neurons with second-order neurons; different intensities of local inhibition in gustatory circuits; or any combination of these processes^40–42^. For temperature detection, although much less is known at the moment, different sensitivities could also be due to different types/qualities of TRP channels at the periphery (possibly involving HsTRPA)^43,44^ or to different neuron/circuit organizations at more central levels. Physiologically, inter-individual differences in appetitive or aversive sensitivities may also arise due to different neuromodulator levels. Biogenic amines, for instance, could be involved, as they have an orchestral function in the modulation of insect behavior^45^, most prominently in social insects^46^. The biogenic amines octopamine and dopamine play an *instructive* role in appetitive and aversive learning in bees, by representing respectively the appetitive and the aversive US in the brain^17,47,48^. This instructive role is however limited to the associative learning event, but biogenic amines are thought to have wider-ranging roles, including the modulation of bees’ responsiveness to sensory stimuli^49–52^. It has been observed for instance that octopamine, tyramine and dopamine can modulate sucrose responsiveness^49,51^. While injections of octopamine or tyramine *increase* bees’ sucrose responsiveness, injections of dopamine or a dopamine receptor agonist *decrease* it. The effect of biogenic amines on sting responsiveness to thermal stimuli, as used here, has not been tested yet. However, recent data using pharmacological injections of amine receptor antagonists suggested that both serotonin and dopamine can reduce bees’ responsiveness to an electric shock, while octopamine has no effect^50^. In our case, different individuals may display discrepancies in biogenic amine levels which would translate into differences in their sensitivity to sucrose and to heat stimuli. It would thus be especially interesting in future work to evaluate whether our bees with lower sucrose responsiveness show lower octopamine/tyramine levels, and bees with lower heat responsiveness show higher serotonin levels, as predicted by the studies above^49,50^.

### The appetitive/aversive sensory trade-off

The most important finding of our study is that the sensitivities of bees toward appetitive and aversive stimuli are under the influence of a genotypic trade-off. Bees with a high sensitivity to sucrose tended to show a low sensitivity to thermal stimuli, and vice versa. How does such a trade-off come about? In theory, the hedonic trade-off could follow a monogenic determinism, if the responsible gene displayed a high allelic polymorphism and had pleiotropic effects on both appetitive and aversive sensitivities. In this case, different patrilines would carry different alleles, giving rise to a continuous distribution of hedonic biases, from aversively-biased to appetitively-biased individuals, as observed here. For instance, a gene that would act positively on both tyramine (or octopamine) and serotonin levels could act on the hedonic bias. Increasing the levels of both amines would give rise to appetitively-biased bees (by increasing sucrose responsiveness and decreasing thermal responsiveness), while decreasing the levels of both amines would favor aversively-biased bees^49,50^. It is however much more likely that the hedonic trade-off is under polygenic influence, as many quantitative traits actually depend on intricate networks of interacting genes^53,54^. The genes responsible for the hedonic trade-off we have described may be related to previous QTL (Quantitative Trait Loci) identified in the honeybee genome and involved in variations of foraging (*pln1-4*,^55–57^) or defensive behaviors (*sting1-3*,^57,58^). Interestingly, genes associated with biogenic amine signaling have been identified within these QTL regions^57^. Thus, *pln2* contains *AmTyr1*, coding for the honeybee tyramine receptor^59^ and *sting3* contains *Am5HT*_7_, coding for one of the honeybee serotonin receptors^60^. Alternatively or in addition to the hypothesis of different biogenic amine levels mentioned above, bees’ appetitive and aversive responsiveness may depend on different allelic forms of tyramine and serotonin receptors respectively. In any case, for the trade-off to appear, the genes supporting appetitive and aversive responsiveness need to engage in epistatic interactions. Genes supporting a high sucrose sensitivity would negatively affect processes involved in heat sensitivity, and vice versa. Such epistasis could happen at several levels, from direct gene interactions by transcription factors^61^ or RNA interference processes^62,63^, or more indirectly, from interactions of the products of these gene with biosynthetic pathways and/or developmental processes. Such epistatic interactions are expected to be highly complex and intensive work will be needed to understand the genotypic trade-off on a functional level. The present study thus paves the way for a long-term exploration of epistatic interactions between aversive and appetitive genetic pathways.

### The hedonic trade-off at the evolutionary level

It has been suggested that social insect colonies with a high genetic diversity are more adaptable than low-diversity colonies^64,65^. Similarly, colonies with a high proportion of specialized individuals are thought to be more efficient that homogeneous colonies^30,66,67^. The hedonic specialization of patrilines, as demonstrated here, may be an adaptive mechanism for honeybees, allowing them to respond efficiently to the ecological constraints surrounding the colony, both in terms of food availability and of prevalence of potential predators and parasites. At the individual level, the trade-off suggests that a high sensitivity and high learning performances in one hedonic modality come at the cost of a lower sensitivity and lower learning performances in the other. At the colony level, however, ecological success and fitness may be more related to the simultaneous presence of both strongly appetitively-biased and strongly aversively-biased workers. Therefore, the hedonic trade-off may have been selected over evolutionary times. Different ratios of appetitive-biased*/*aversive-biased workers may be adapted to different environmental conditions, with for instance a better fitness for a higher proportion of appetitively oriented individuals in high-resource sites and a higher proportion of aversively oriented individuals in low-resource sites. However, the long term interest of the species would be to maintain a good balance of both types of individuals for adapting to local conditions. Honeybees are characterized by a monogynous polyandrous mating system, with typically as many as 15–20 males inseminating a queen^29^. This high polyandry increases the probability of sampling alleles from the whole genetic diversity in the population and maintaining rare alleles that may not be currently adapted but may be beneficial in the future^68^. A next step for understanding the evolution of the hedonic trade-off and possible adaptations to local conditions would be to measure the hedonic bias in workers from colonies with a common genetic origin but maintained over generations in high- or low-resource sites. We expect to find in these colonies different proportions of appetitively- and aversively-biased individuals. Such adaptations of the hedonic bias may be a basis for the observation that, at the population level, hives with a high foraging activity display low defense responses and vice versa^19^.

In conclusion, we found a trade-off in honeybees’ sensitivity and learning abilities between appetitive and aversive hedonic modalities, which depends on a genotypic determinism. Such trade-off may be instrumental for efficient task allocation within the colony and for its rapid adaptation to local environmental conditions. On a proximal level, future work will need to focus on the epistatic effects giving rise to this trade-off. On a more distal level, studying how bees adapt this trade-off with local conditions may help understand its possible beneficial effect for bees’ ecological success.

## Methods

### Animals

Age-controlled bees were used in the experiments to avoid any potential impact of age on bees’ behavioral responses. Thirteen-14 day-old bee workers were obtained from two hives on the CNRS campus Gif-sur-Yvette. Shortly, a comb with capped brood, close to emergence was taken from the hive and all adult bees were gently brushed aside. The comb was then placed in a closed box in an incubator at 34 °C. On the next day, newly emerged bees were painted with a two-color code (Posca, France) and then placed back into the hive. Thirteen days later, the bees were taken from the hive and used in the behavioral experiments. At this age, honey bees usually start to perform tasks outside the hive such as guarding or foraging^37^.

After chilling on ice, 16 individuals were harnessed in a metal holder as described in Junca *et al*.^16^. With this holding procedure, both sting- and proboscis extension could be clearly monitored. Bees were fed with 5 µl of sucrose solution (50% w/w) every morning and evening to keep them in a good condition for the two experimental days and were conserved in a dark and humid box between experiments. One group of 16 bees was tested over two days. Four experimental procedures were carried out on these individuals according to the following schedule: half of the bees were subjected to the measure of *sucrose responsiveness* followed by *appetitive conditioning* on the first day and to the measure of *heat responsiveness* followed by *aversive conditioning* on the second day. For the other half, the two experimental days were swapped. At the end of the second day, all bees were placed in individual Eppendorf tubes filled with 96% ethanol solution for microsatellite analysis.

### Bees’ responsiveness to temperature and sucrose stimuli

Once mounted, bees were placed in a dark and humid box for two hours to avoid any stress. Thermal responsiveness was measured following the procedure of Junca *et al*.^16^. Bees received a succession of six stimulations of increasing temperature (from ambient temperature ~25 °C to 75 °C), in steps of 10 °C. Thermal stimulations were provided by means of a pointed copper cylinder (widest diameter: 6 mm; length: 13 mm), mounted onto the end of a minute soldering iron running at low voltage (HQ-Power, PS1503S). Temperature at the end of the cylinder was controlled using a contact thermometer (Voltcraft, Dot-150). Thermal stimulations alternated with tactile controls, provided as above with an identical unheated probe.

Sucrose responsiveness was measured following the protocol described in Scheiner *et al*.^34^. Bees were presented sucrose solutions of increasing concentration following an exponential progression (0%; 0.1%; 0.3%; 1%; 3%; 10%; 30% w/w). Sucrose stimulations were alternated with water control. Sucrose and water stimulations were provided with a soaked toothpick to the bees’ two antennae simultaneously, and the PER (extension or not of the proboscis) was noted.

In both heat and sucrose responsiveness experiments each trial lasted 38 s. The bee was placed in the holding setup, and left for 20 s before stimulus application started. The sucrose or thermal stimulation lasted for 1 s, and was applied to both antennae for sucrose responsiveness or to the mouthparts for heat responsiveness. The bee was then left in the setup for 17 s and was removed. For a given bee, all stimulations were performed at 10 min intervals.

### Bees’ aversive and appetitive learning performance

On each day, the learning procedure started 1 h after the responsiveness procedure. Learning procedures were identical for appetitive and for aversive conditioning, except for the US used and the behavioral response measured. During appetitive conditioning, the US was a 30% sucrose solution and PER were measured. During aversive conditioning, the US was a 65 °C thermal stimulation to the mouthparts and SER were measured.

Bees were subjected to differential conditioning procedures, in which one odorant (the CS+) was associated with either appetitive or aversive reinforcement (the US), while another odorant was presented without reinforcement (the CS−). Two pairs of odorants were chosen according to Guerrieri *et al*.^69^, in such a way that all odorants were well differentiated from each other by bees. For each bee, one odorant pair was used for aversive conditioning while the other was used for appetitive conditioning. To avoid producing a high number of subgroups, within each odorant pair, one odorant was used as CS+ while the other was used as CS−. The two pairs of odors were: (1) 1-nonanol (CS+) and 2-heptanol (CS−); (2) hexanal (CS+) and 2-octanone (CS−) (Sigma Aldrich, Deisenhofen, Germany). Five μl of pure odorant were applied onto a 1 cm² piece of filter paper which was transferred into a 20 ml syringe (Terumo) allowing manual odorant delivery to the antennae. Half of the bees were conditioned with odorant pair 1 for aversive conditioning and odorant pair 2 for appetitive conditioning, and vice versa for the other half.

Each conditioning procedure was composed of 16 trials (8 reinforced and 8 non-reinforced) in a pseudo-random sequence (e.g. ABBABAAB) starting with odorant A or B in a balanced way. The inter-trial interval (ITI) was 10 min. Each conditioning trial lasted 38 s. The bee was placed in the stimulation site in front of the air extractor, and left for 18 s before being exposed to the odorant paired with the US. Each odorant (CS+ or CS−) was delivered manually for 4 s. The thermal stimulus started 3 s after odorant onset and finished with the odorant (1 s temperature stimulation). The bee was then left in the setup for 14 s and was then removed.

### Determination of patriline origin

Patriline determination was carried out by genotyping microsatellite areas conserved in the bees’ genome. Microsatellites are non-coding DNA fragments, made of repeated pairs (duo) or triplets (or more) of nucleotides. Sizes of microsatellites are conserved in bees’ offspring (patrilines) like alleles. To precisely determine the patriline origin of each bee, 12 loci were amplified^70^.

DNA was extracted using the 10% Chelex method adapted for squashed bee head tissues^71^. The head of the bee was cut off and placed in an Eppendorf tube with an iron marble. The tube was then placed into a grinder (Retsch MM301). Once the head crushed, 600 µl of 10% Chelex (BioRad) at 60 °C were added. Composed of micromarble, the Chelex chelates impurities and ions which could interfere with the following PCR. Then, 18 µl of proteinase K were added and after 1 h digestion at 50 °C in a heating block, the tubes were placed 30 min at 90 °C to remove proteinase K. The iron marbles were then removed and the solutions centrifugated for 10 min at 12000 rpm. They were then conserved in a freezer (−20 °C).

Microsatellites amplifications were performed using 3 different multiplexes, which allowed analyzing several loci simultaneously. Multiplex 1 was composed of loci A88, A28, A24, Ap55 and A66. Multiplex 2 was composed of loci A113, A7, Ap43 and Ap81. Multiplex 3 analyzed loci Ap33, A43, A8. A multiplex contains pure water, buffer (Promega), Bovine serum albumin (BSA; Sigma Aldrich) and Taq polymerase which allows replicating the fragments of interest. In a PCR dish, 1 µL of non-diluted DNA and 9 µL of the chosen plex were deposited. The time spent in the thermocycler (Biometra, UNO-thermobloc) was calibrated for each multiplex, depending on the primers used. For genotyping in the sequencer, a mix of Rox350 and Formamide was added to the PCR product. DNA fragments were identified using an ABI 3130 Genetic Analyzer and the Genscan analysis software (version 3.7.1). Allelic sizes were labeled using Genemapper 4.1. Allele nomenclature was standardized using reference samples^72–74^.

The multilocus genotype of the queen was verified, using the Colony 1.2 program^75^. The program analyzes haplo-diploid systems based on the expression of codominant genetic markers, such as DNA microsatellites. It calculates the probabilities of all possible queen genotypes, based on the observed allele frequencies in the population. Paternal alleles for each worker were then characterized after subtracting the queen’s allele from each worker’s genotype. Workers were considered as belonging to the same patriline when the same alleles were shared over all (12) analyzed loci. Patrilines with more than 8 individuals were used in the analyses. This threshold yielded 11 patrilines in total, nine from colony 1 [patrilines n°1–8 and 11 in Fig. 6] and two from colony 2 [patrilines 9 and 10 in Fig. 6]). Although maternal genetic effects may also play a role in the variability between these groups (because of the two colonies), we stuck to the term patriline throughout for commodity.

### Statistical analysis

All recorded data were dichotomous, with a sting or proboscis extension being recorded as 1 and a non-extension as 0. Over all analyses, bees which did not respond during either one of the responsiveness experiments were excluded from the analysis, as they were considered as not appetitively or aversively motivated enough to learn in the following conditioning experiments.

We calculated for each bee its *thermal responsiveness score* (from 1 to 6) and *sucrose responsiveness score* (from 1 to 7) by counting the number of times it responded to the thermal or sucrose stimulus of increasing intensities. Higher scores indicate bees that started to respond at lower temperatures or sucrose concentrations, and are thus more sensitive to temperature or sucrose respectively. In the same manner, two learning performance scores were calculated. For the *aversive* and *appetitive learning scores*, we counted the number of times bees responded to the reinforced odorant (CS+). A higher score indicated a good learner, which quickly associated the CS+ with reinforcement.

Data from both colonies (colony 1, n = 157; colony 2, n = 69) and for both subgroups which received different arrangements of odorant pairs for aversive/appetitive tasks (appetitive conditioning with 1-nonanol/2-heptanol, n = 108; with 1-hexanol/2-octanone, n = 118 – vice versa for aversive conditioning) were pooled. This was possible because initial statistical analyses indicated that these subgroups had no differential effects on bees’ response threshold and learning scores. While the *colony* had a general effect on bees’ average score to the four tasks (Repeated-measure ANOVA, F_1,222_ = 8.23, p < 0.01), there was no interaction between *colony* and *task* (F_3,666_ = 2.16, NS). Thus, while scores were generally higher in one colony than in the other, this did not affect the relative scores of the bees in the four tasks. Concerning the subgroups based on the odorant pairs used for each task, we found no general effect on bees’ scores in the four tasks (RM-ANOVA, F_1,222_ = 0.90, NS) and no interaction between *odorant pair* and *task* (F_3,666_ = 1.24, NS). Second, when taking into account both the valence (appetitive *vs* aversive) and the type of task (threshold *vs* conditioning) for the scores (2 repeated measures), we found no significant *valence* x *task* x *colony* interaction (RM-ANOVA, F_1,222_ = 0.62, NS) and no *valence* x *task* x *odorant* pair interaction (RM-ANOVA, F_1,222_ = 0.88, NS).

To analyze *thermal* and *sucrose responsiveness* curves or *appetitive and aversive conditioning* curves, we used repeated measure ANOVAs with stimulus (either thermal (sucrose) vs tactile (water), or CS+ vs CS−) and trial as repeated factors. Monte Carlo studies have shown that it is permissible to use ANOVA on dichotomous data only under controlled conditions, which are met in these experiments^76^.

A correlative approach was chosen to analyze relationships between responsiveness and learning performances within or across hedonic modalities at the individual and at the patriline levels. For studying correlations at the individual level, bees were grouped by heat responsiveness score and their average learning performance scores were calculated, thus allowing a clear representation of the relationship between the two variables. At the patriline level, bees thermal and sucrose responsiveness scores and aversive and appetitive learning scores were averaged for each patriline. Correlations were assessed by calculating the Spearman correlation coefficient. To further reveal positive or negative relationships among response scores, Factor Analyses (FA) were used. These analyses were complemented with a cluster analysis based on Euclidian distances between patrilines’ behavioral responses in order to highlight putative groupings of patrilines exhibiting similar hedonic biases. All data were analyzed with STATISTICA V5.5 and V10.0 (StatSoft, Tulsa, USA). In the graphs, data are presented as the mean ± SEM.

## Data Availability

All data are available upon request from the corresponding author.
